# Coexistence Tumor of Craniopharyngioma and Pituitary Neuroendocrine Tumor: A Case Report and Literature Review

**DOI:** 10.7759/cureus.93606

**Published:** 2025-09-30

**Authors:** Yohei Nounaka, Fumihiro Matano, Koshiro Isayama, Keiko Tomiyama, Chie Inomoto, Robert Y Osamura, Shigeyuki Tahara, Yasuo Murai

**Affiliations:** 1 Neurological Surgery, Nippon Medical School, Tokyo, JPN; 2 Neurosurgery, Nippon Medical School Hospital, Tokyo, JPN; 3 Endocrinology, Diabetes and Metabolism, Nippon Medical School Hospital, Tokyo, JPN; 4 Pathology, Tokai University School of Medicine, Isehara City, JPN; 5 Pathology, Nippon Koukan Hospital, Kawasaki, JPN; 6 Neurological Surgery, Nippon Medical School Musashikosugi Hospital, Kawasaki, JPN

**Keywords:** coexistence tumor, craniopharyngioma surgery, endoscopic endonasal transsphenoidal surgery, growth hormone therapy, pituitary endocrine tumor

## Abstract

Acromegaly most commonly results from excess growth hormone (GH) produced by pituitary neuroendocrine tumors (PitNETs). Craniopharyngioma (CP) is an uncommon suprasellar tumor characterized by cystic change and calcification. The true coexistence of PitNET and CP is rare and poses diagnostic and operative challenges, particularly when lesions are spatially separated.

A 72-year-old man was referred for a pituitary mass and clinical features suggestive of acromegaly (enlarged extremities, prominent supraorbital ridge, and jaw enlargement) without headache or visual complaints. MRI demonstrated two distinct lesions: a heterogeneous, mainly cystic suprasellar mass measuring 2.2×1.2×1.2 cm compressing the optic chiasm and extending toward the third ventricle, and a separate 1.7×1.2×1.1 cm intrasellar lesion extending into the sphenoid sinus. CT showed calcification within the suprasellar lesion. Baseline hormones revealed elevated GH (7.60 ng/mL) and IGF-1 (388 ng/mL; +5.7 SD) with inadequate GH suppression on oral glucose tolerance testing (nadir GH 2.68 ng/mL). An endoscopic endonasal transsphenoidal approach was undertaken. Intraoperatively, a white, soft intrasellar tumor breaching the dura and protruding into the sphenoid sinus was removed, and a separate, highly calcified suprasellar tumor was internally decompressed and dissected free; no macroscopic continuity was identified. Histopathology showed a densely granulated mixed somatotroph-lactotroph PitNET (immunoreactive for GH, prolactin, Pit-1, and alpha subunit; CAM5.2 perinuclear pattern <70%; Ki-67 <1%) and an adamantinomatous CP with wet keratin and calcified nests. Postoperatively, GH fell to 0.36 ng/mL and IGF-1 to 166 ng/mL (+1.2 SD). Transient diabetes insipidus occurred and was controlled with desmopressin. The patient was discharged without new neurological deficits, and an MRI at four months showed no recurrence.

This case underscores the value of recognizing “separated” coexistence of CP and PitNET, in which discrepant imaging features (calcified, cystic suprasellar mass versus enhancing intrasellar lesion) can suggest dual pathology before surgery. Clear preoperative identification facilitates tailored resection strategies, allows focused manipulation of each lesion, and helps balance the competing priorities of gross-total removal and preservation of pituitary-hypothalamic function. Early biochemical remission of acromegaly and an uncomplicated radiographic course support the efficacy and safety of an endoscopic endonasal approach in selected patients.

When intra- and suprasellar lesions demonstrate discordant radiologic characteristics in a patient with biochemical acromegaly, concomitant CP and PitNET should be considered. Distinguishing separated from admixed disease preoperatively can guide operative planning and reduce morbidity. Targeted endoscopic resection achieved prompt hormonal normalization and radiographic disease control in this patient, emphasizing the importance of individualized, anatomy-driven management and vigilant postoperative endocrine and imaging follow-up.

## Introduction

Acromegaly is caused by the hypersecretion of growth hormone (GH). GH-producing pituitary neuroendocrine tumors (PitNETs) are found in 95% of patients with acromegaly [1]. The National Database of Health Insurance Claims and Specific Health Checkups of Japan reported that the average annual prevalence in 2015-2017 was 9.2 cases per 100,000 in the prevalence cohort, and the average annual incidence in 2013-2017 was 0.49 cases per 100,000 in the incidence and comorbidity cohort [2].

Craniopharyngioma (CP) is characterized by neuroradiological features, such as solid parts, cyst walls, and calcification. CP accounts for 1.2-4.6% of all intracranial tumors, with an annual incidence of 0.5-2.5 cases per 1 million cases and various clinical manifestations including increased intracranial pressure, endocrine disorders, and visual disturbances [3].

Multiple endocrine neoplasia type 1 (MEN1) exemplifies the co-occurrence of PitNETs with other endocrine neoplasms - most commonly of the parathyroid and pancreas. Although such extra-pituitary associations are well documented, concomitance of a PitNET with a craniopharyngioma is rare [1].

Intracranial coexisting tumors are diseases in which two varying tumors coexist; the most common tumor types are benign meningiomas and glioblastomas, but fewer than 100 cases were reported in 2018 [4].

We encountered a rare case of coexisting PitNET and craniopharyngioma. Because their clinical and investigative findings often overlap, this constellation can materially influence surgical planning and timing. Accordingly, we conducted a literature review comparing patient demographics, clinical presentations, endocrine profiles, imaging discriminators, therapeutic strategies and outcomes, and postoperative complications, with the objective of delineating practical, practice-oriented considerations for routine care.

## Case presentation

**Figure 1:**
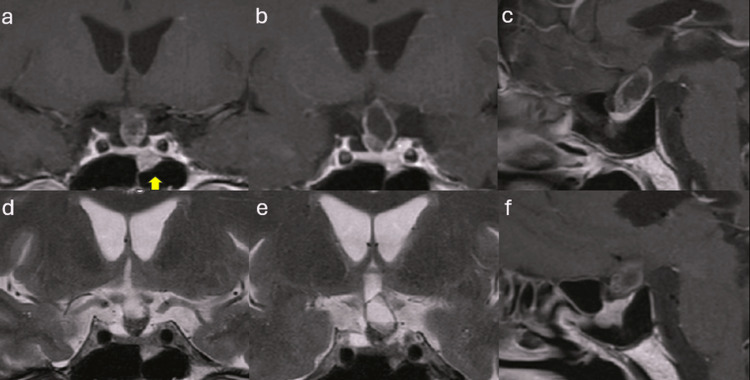
Preoperative brain MRI: coronal (a, b) and sagittal (c, f) T1-weighted, contrast-enhanced sequences, and coronal (d, e) T2-weighted, non-contrast sequences. The arrowhead reveals the pituitary neuroendocrine tumor (PitNET), and the suprasellar cystic lesion illustrates the craniopharyngioma.

A 72-year-old man with a pituitary tumor was referred to our department. The patient had a history of mesopharyngeal carcinoma treated with chemoradiotherapy and was in remission. The patient had no relevant family history, and their clinical features included enlarged hands and feet, a prominent supraorbital ridge, and a large nose and jaw with suspected acromegaly. No headaches or visual disturbances were noted. Magnetic resonance imaging (MRI) disclosed a suprasellar heterogeneous, mainly cystic, 2.2×1.2×1.2-cm mass compressing the optic chiasm and expanding to the third ventricle without causing hydrocephalus, and the lesion outside the sella turcica, a 1.7×1.2×1.1-cm mass extending to the sphenoid sinus. Gadolinium-enhanced MRI of the patient’s head revealed heterogeneous contrast enhancement of the suprasellar tumor and homogeneous contrast enhancement of the intrasellar tumor (Figure 1).

Computed tomography disclosed calcification of the suprasellar lesion. The findings of the pituitary-related hormonal assay were as follows: thyroid-stimulation hormone = 0.77 μIU/mL (normal range: 0.34-3.88 μIU/mL), free triiodothyronine = 3.18 pg/mL (normal range: 2.13-4.07 pg/mL), free thyroxine = 1.01 ng/dL (normal range: 0.95-1.74 ng/dL), adrenocorticotropic hormone = 30.1 pg/mL (normal range: 7.2-63.3 pg/mL), cortisol = 11.0 μg/dL (normal range: 5.3-22.5 μg/dL), prolactin = 12.9 ng/mL (normal range: 3.7-16.3 ng/mL), GH = 7.60 ng/mL (normal range: 0-2.47 ng/mL), insulin-like growth factor-1 (IGF-1) = 388 ng/mL (+5.7 standard deviation (SD), normal range: 84-239 ng/mL), luteinizing hormone = 3.7 mIU/mL (normal range: 0.1-8.7 mIU/mL), and follicle-stimulating hormone = 9.8 mIU/mL (normal range: 2.0-8.3 mIU/mL) (Table 1). GH and IGF-1 levels were elevated above the baseline values. An oral glucose tolerance test indicated insufficient GH level suppression (nadir GH: 2.68 ng/mL, normal values: <0.4 ng/mL).

**Table 1:** Pituitary-related hormonal assay

Parameter	Values found in the patient	Normal reference value
Thyroid-stimulation hormone	0.77 μIU/mL	0.34–3.88 μIU/mL
Free triiodothyronine	3.18 pg/mL	2.13–4.07 pg/mL
Free thyroxine	1.01 ng/dL	0.95–1.74 ng/dL
Adrenocorticotropic hormone	30.1 pg/mL	7.2–63.3 pg/mL
Cortisol	11.0 μg/dL	5.3–22.5 μg/dL
Prolactin	12.9 ng/mL	3.7–16.3 ng/mL
Growth hormone	7.60 ng/mL	0–2.47 ng/mL
Insulin-like growth factor-1	388 ng/mL	84–239 ng/mL
Luteinizing hormone	3.7 mIU/mL	0.1–8.7 mIU/mL
Follicle-stimulating hormone	9.8 mIU/mL	2.0–8.3 mIU/mL

**Figure 2:**
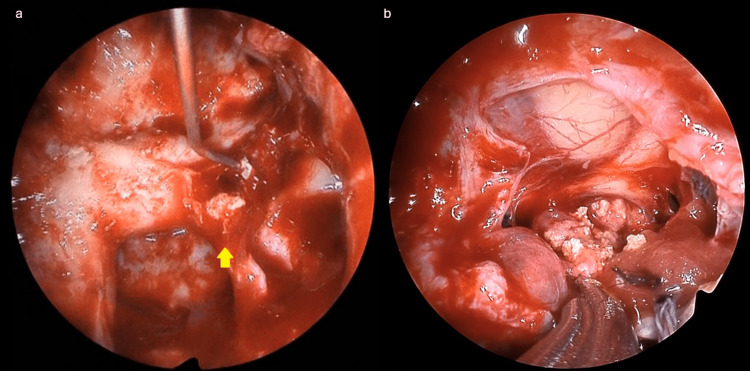
Intraoperative visualization (a) Arrowhead illustrates a white, soft, pituitary neuroendocrine tumor (PitNET). (b) The white calcified tumor is a craniopharyngioma.

We considered the possibility of a functioning PitNET and CP and planned endoscopic endonasal transsphenoidal surgery. The two tumors were distant from each other, and the possibility that they varied was considered in treating this case. Intraoperative findings revealed that the GH-producing PitNET was situated in the left inferior part of the internal sella, breached the dura, and protruded into the sphenoid sinus. The tumor was white and soft (Figure 2a). A dural incision showed another highly calcified tumor, which was considered most likely a CP (Figure 2b).

The tumor extended from below the optic chiasm to the posterior pituitary and was removed via internal decompression and dissection of the surrounding tissue. No obvious continuity was noted between the two tumors.

**Figure 3:**
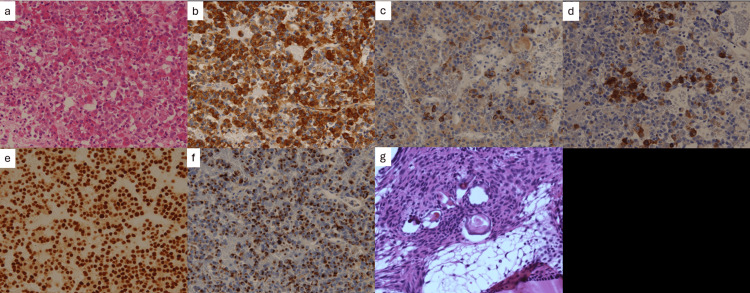
Pathological findings of the tumor (a) Hematoxylin and eosin (HE) staining reveals that the intrasellar tumor was composed of eosinophilic cells in a trabecular pattern with a rounded and slightly enlarged nucleus. Immunostaining was positive for growth hormone (b), prolactin (c), alpha subunit (d), Pit-1 (e), and CAM5.2 (f). (g) The suprasellar tumor demonstrated pseudostratified ciliated epithelium and palisaded basal layer of small cells with darkly staining nuclei and little cytoplasm, with wet keratin and calcified nests (HE stain).

A histopathological assessment was conducted for each tumor. Hematoxylin and eosin (HE) staining showed that the intrasellar tumor was composed of eosinophilic cells in a trabecular pattern with rounded and slightly enlarged nuclei (Figure 3a). Immunostaining was positive for GH, prolactin, alpha subunit, and Pit-1 (Figure 3b-3e). CAM5.2 was positive for perinuclear but accounted for <70% of the total (Figure 3f). The pathological findings resulted in the diagnosis of a densely granulated mixed somatotroph-lactotroph PitNET. The Ki67 proliferation index was <1%. HE staining revealed that the suprasellar tumor demonstrated a pseudostratified ciliated epithelium and a palisaded basal layer of small cells with darkly stained nuclei and little cytoplasm, with wet keratin and calcified nests. This resulted in the diagnosis of adamantinomatous CP (Figure 3g).

The postoperative hormonal assay revealed a decrease in GH and IGF-1, demonstrating a decrease in the baseline values (GH = 0.36 ng/mL, IGF-1 = 166 ng/mL (+1.2 SD)). The patient developed postoperative diabetes insipidus, which was controlled with desmopressin acetate hydrate. The patient was discharged without substantial complications.

Four months after surgery, there were no new symptoms, and the patient was followed up with MRI without any recurrence.

## Discussion

We report a case of PitNET combined with CP. A literature review was performed using all English and Japanese articles on relevant studies and case reports published before April 2024, which were searched using the PubMed and Medical Journal databases. Keywords included “pituitary adenoma,” “pituitary neuroendocrine tumor,” or “PitNET” and “craniopharyngioma.” Clinical studies of this disease were selected, including references that could provide individual patients’ clinical information included in these case reports and studies. PitNET cases with CP are rare, with only 21 previously reported cases (Table 2) [4-20].

**Table 2:** Literature review of pituitary neuroendocrine tumors with craniopharyngiomas PitNET: pituitary neuroendocrine tumor; CP: craniopharyngioma; M: male; F: female; TCS: transcranial surgery; TSS: transsphenoidal surgery; CSF: cerebrospinal fluid; IGF-1: insulin-like growth factor-1; PRL: prolactin; FSH: follicle-stimulating hormone; GH: growth hormone; ACTH: adrenocorticotropic hormone. Items that were not described in the reviewed cases were defined as not described (ND).

Author	Reference	Year	Age	Sex	Symptom	Size (mm)		Endocrinological state	First treatment	Further treatment	Postoperative complication	Pathological type of craniopharyngioma	Pathological type of PitNET	Follow-up (month from the last surgery)	Prognosis
						PitNET	CP								
Prabhakar et al.	[17]	1971	29	M	Visual disturbance, headache, acromegaly	ND	ND	ND	TCS	None	Diabetes insipidus	Adamantinomatous	Somatotroph	4 days	Deceased due to diabetes insipidus
Karavitaki et al.	[13]	2008	50	M	Headache, hypogonadism	12		Mildly high PRL, hypogonadism	TSS	None	None	Adamantinomatous	Gonadotroph	48	Good
Yoshida et al.	[20]	2008	29	M	Atrial fibrillation	46×38×36		High TSH and αsubunit, low GH	TSS	None	Recurrent epistaxis requiring transfusion and arterial embolisation	Adamantinomatous	Thyrotroph	ND	ND
Sargis et al.	[18]	2009	59	M	Visual disturbance	33×33×34		High FSH	TCS	None	Transient diabetes insipidus	Adamantinomatous	Gonadotroph	ND	Good
Moshkin et al.	[16]	2009	8	M	None	20		Mildly high PRL, hypogonadism, hypothyroidism	TCS	None	None	Adamantinomatous	Non-functioning	10	Good
Gokden and Mrak	[10]	2009	47	M	Visual disturbance, headache	58×54		Normal	TSS	None	None	Adamantinomatous	Non-functioning	12	Good
El-Bilbeisi et al.	[7]	2010	41	F	Visual disturbance, headache, acromegaly, amenorrhea	ND	ND	Hypogonadism, mildly high PRL, hypothyroidism, high GH/IGF-1	TSS	TCS (18 years)	None	Adamantinomatous	Somatotroph	6	Good
Guaraldi et al.	[11]	2013	27	F	Amenorrhea, galactorrhea	4	19×13	Mildly high PRL	TSS	TCS (11 years)	None	Papillary	Lactotroph	6	Good
Jin et al.	[12]	2013	47	F	Visual disturbance, headache	ND	ND	High PRL and ACTH	TSS	TCS (11 m)	Hyponatremia, transient diabetes insipidus	Adamantinomatous	Non-functioning	24	Good
Finzi et al.	[8]	2014	75	F	Diplopia	30×18×18		Mildly high PRL	TSS	None	None	Adamantinomatous	Corticotroph	10	Good
Bhatoe et al.	[5]	2016	35	M	Visual disturbance, headache, seizures, acromegaly	ND	32×49×43	High GH	TCS	None	None	Adamantinomatous	Somatotroph	36	Good (visual disturbance)
Fountas et al.	[9]	2018	65	M	Visual disturbance, headache, fatigue, acromegaly	12×4	19×20×19	Hypogonadism, mildly high PRL, high IGF-1	TSS	Radiation, somatostatin analogue	None	Adamantinomatous	Somatotroph	12	Good (treatment of lung cancer)
Snyder et al.	[19]	2019	49	F	Visual disturbance, headache	15×13×20	12×12×15	Hypogonadism	TSS	TCS	CSF leakage (repair), meningitis	Adamantinomatous	Corticotroph	13	Good
Miyazaki et al.	[15]	2019	48	M	Visual disturbance, memory disorder	ND	ND	Mildly high PRL	TCS	TSS (2 months), gamma knife surgery	None	Adamantinomatous	Non-functioning	120	Good
Bteich et al.	[6]	2020	35	M	Visual disturbance, headache	ND	ND	Hypogonadism	TSS	None	None	Papillary	Non-functioning	6	Good
Hasegawa et al.	[4]	2021	51	M	Visual disturbance, fatigue, headache	27×25×35		Hypocortisolism, hypothyroidism	TSS	TSS (27 months)	None	Adamantinomatous	Gonadotroph	42	Good (cystic recurrence)
Kikuta et al.	[14]	2023	54	M	None	16	2	Mildly high PRL and FSH	TSS	TSS (4 years)	Diabetes insipidus	Adamantinomatous	Thyrotroph	ND	Good (treatment of Graves’ disease)
Present case		2024	71	M	Acromegaly	17×12×11	22×12×12	High GH	TSS	None	Diabetes insipidus	Adamantinomatous	Somatotroph	3	Good

Descriptive summary

Across 21 reported cases of concomitant PitNET and CP, the mean age was 47 years (range: 8-75), and 76.2% were male (16/21). Visual disturbance (66.7%), headache (52.4%), and endocrine abnormalities (42.9%) predominated; initial surgery was transsphenoidal in 71.4% and transcranial in 28.6%, with 52.4% requiring additional treatment and postoperative diabetes insipidus in 23.8%. Favorable outcomes were noted in 85% (17/20 with available outcome data), with three deaths reported (cardiac arrest, pulmonary embolism, and diabetes insipidus).

**Figure 4:**
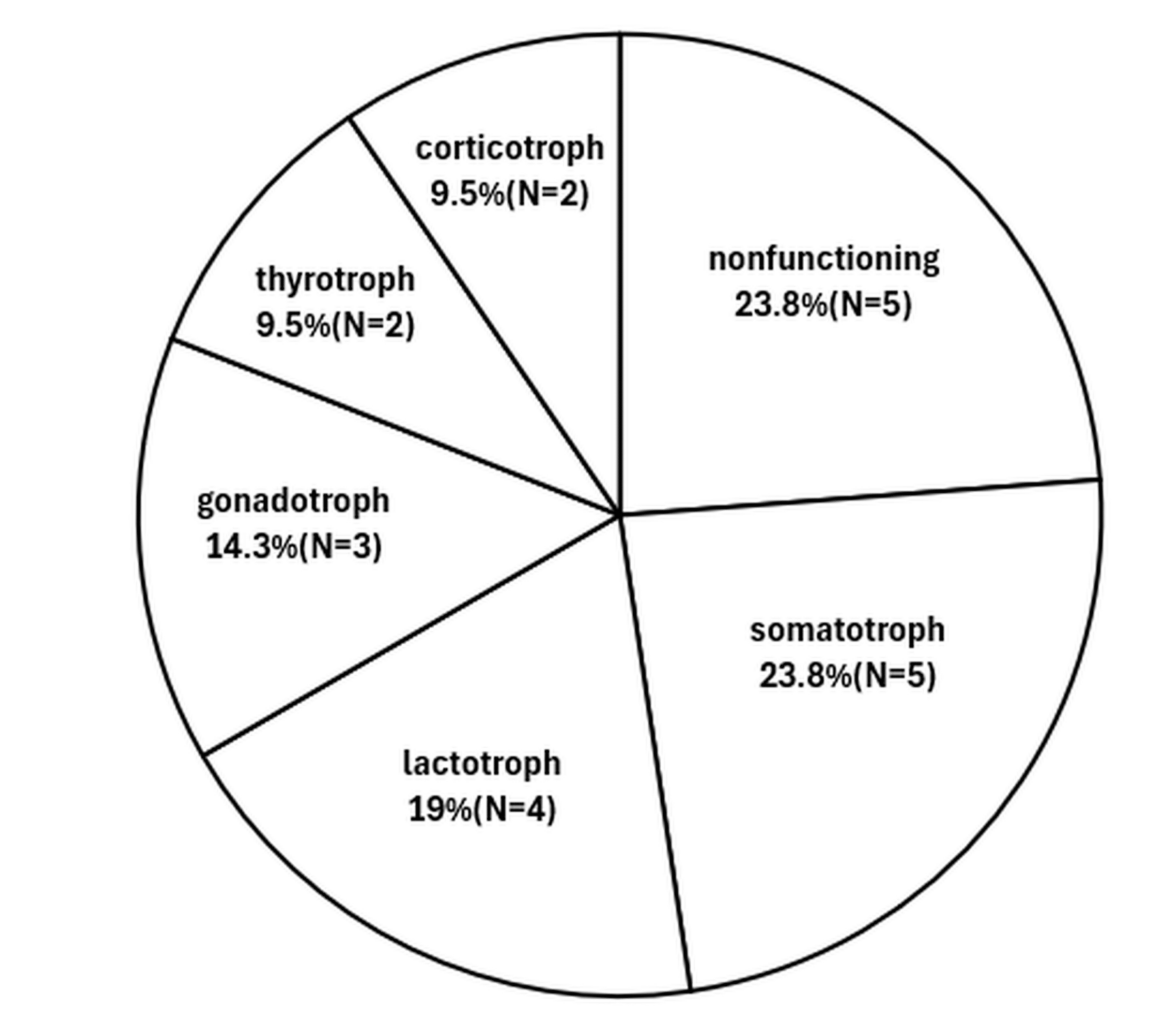
Frequency of pituitary neuroendocrine tumors (PitNETs) with craniopharyngioma classified according to endocrinological function.

The mean patient age was 47 (8-75) years, with 16 cases (76.2%) in men and five (23.8%) in women. Clinical symptoms included visual disturbance in 14 patients (66.7%), headache in 11 (52.4%), endocrine disorder in nine (42.9%), memory disorder in two (9.5%), diplopia in one (4.8%), and no symptoms in one (4.8%). The initial treatment was transsphenoidal surgery in 15 patients (71.4%) and transcranial surgery in six patients (28.6%). Additional treatment was performed in 11 (52.4%) patients: transcranial surgery in seven (33.3%), transsphenoidal surgery in three (14.3%), and radiotherapy in one (4.8%). The postoperative course was good in 17 patients (85%). Among the remaining three patients (15%), one died of cardiac arrest, one of pulmonary embolism, and one of diabetes insipidus. The pathological CP type was adamantinomatous in 19 cases (90.5%) and papillary in two (9.5%). The pathological PitNET type was non-functioning in five cases (23.8%), somatotrophic in five (23.8%), lactotrophic in four (19.0%), gonadotrophic in three (14.3%), thyrotrophic in two (9.5%), and corticotrophic in two (9.5%) (Figure 4).

This is the fifth reported case of coexisting acromegaly and CP [5,7,9,17]. El-Bilbeisi et al. reported that endocrine effects from the excess GH/IGF-1 axis in PitNETs may contribute to CP [7]. All patients with somatotroph PitNETs and CP had acromegaly, and four out of five (80%) exhibited visual disturbances. Three and two patients underwent transsphenoidal and transcranial surgeries, respectively. Additional treatment included transsphenoidal surgery followed by radiotherapy and transcranial surgery 18 years later in one case each. The prognosis was good, except for one patient who died owing to diabetes insipidus [7,9].

Among the 21 reported cases, 19 patients were symptomatic and underwent surgical treatment. Two patients were asymptomatic but proceeded to surgery because the tumors compressed the optic chiasm. In one report by Moshkin et al., the lesion was initially identified and then observed for four years, underscoring the importance of regular surveillance to assess tumor growth even in asymptomatic individuals [16].

Hasegawa et al. reported that coexisting tumors are divided into admixed and separated types. Admixed tumors are removed in one mass, whereas separated tumors require individual tumor identification and different removal strategies [4]. Although predicting tumor coexistence preoperatively from both imaging findings in the admixed type is difficult, predicting tumor coexistence in the separated type may be possible [15]. In this case, the tumors with different imaging findings were separately localized; therefore, surgery was conducted after preoperatively predicting the possible presence of various tumor types.

From an embryologic standpoint, the anterior pituitary and CP share a common Rathke’s pouch origin, arising from oral ectoderm that ascends toward the infundibulum; residual epithelial rests can persist along the sellar-suprasellar axis, creating an anatomic continuum on which dual pathologies may develop [3,4]. Coexistence appears as either collision lesions (adjacent but histologically separate tumors) or composite/admixed lesions (intermixed or divergently differentiated components within one mass) [4,8,10,12,20]. Reports of squamous metaplasia or transitional zones between adenohypophyseal tissue and adamantinomatous components support the latter mechanism in selected cases [8,10,20], whereas other series emphasize clearly separated tumors consistent with independent ontogeny within the same developmental corridor [4,12,15]. Clinically, appreciating this anatomic and developmental continuity helps interpret complex parasellar imaging, prioritize identification of the normal gland and stalk, and tailor operative routes to the likely admixed vs separated configuration to optimize resection while minimizing endocrine morbidity [4,12].

Postoperative enuresis was observed in five cases (23.8%) as a postoperative complication, including the present case, and the enuresis incidence was lower than that in the typical CP postoperative period. This may be attributed to fewer cases being completely removed.

In this review, 52.4% of cases required additional treatment after the initial intervention, highlighting the importance of rigorous postoperative surveillance. Moreover, the interval from initial treatment to retreatment ranged from two months to 18 years, underscoring the need for long-term, stringent follow-up [4,7,11,12,14,15,19]. Aiming for complete (gross-total) resection to prevent recurrence - even while balancing the risk of uropathy - and maintaining strict, long-term surveillance is crucial [4].

## Conclusions

In patients with biochemical acromegaly and discordant intra- and suprasellar imaging features, the possibility of coexisting PitNET and craniopharyngioma should be actively considered. Preoperative recognition of “separated” lesions enables an anatomy-driven surgical plan that targets each tumor while balancing the goals of gross-total resection and preservation of hypothalamic-pituitary function. In our patient, an endoscopic endonasal approach achieved prompt biochemical remission and radiographic control, with transient diabetes insipidus managed medically. When two distinct lesions coexist in the parasellar/suprasellar region, imaging interpretation becomes more complex. Precise identification of the normal pituitary gland and stalk, delineation of their spatial relationships to each tumor, and incorporation of these findings into preoperative surgical planning are essential.

This case underscores the diagnostic value of carefully integrating endocrine testing with detailed MRI and CT characteristics to anticipate dual pathology. Clear intraoperative compartmentalization, tailored dissection, and pathology-specific reconstruction are practical steps that can reduce morbidity. Vigilant postoperative endocrine surveillance and interval neuroimaging remain essential to confirm durable tumor control and guide timely intervention if needed.
